# Obstetrics

**Published:** 1861-03

**Authors:** 


### Bi-Monthly Periscope.

### OBSTETRICS.

OPERATION OF OVARIOTOMY; RECOVERY. By *Wm. H. Byford, A.M., M.D.*, Professor of Obstetrics, etc., Medical Department of Lind University.

Miss E., aged fifteen years, from Roseville, Illinois, was brought to me by her parents during May, 1860, to obtain advice respecting an abdominal enlargement of which she was the subject. An examination revealed a multilocular ovarian tumor of large size, occupying nearly the whole abdominal and pelvic cavities, and producing an enlargement exceeding that of pregnancy at full term. The history of the case was very obscure, and the little that was obtained was furnished by Dr. Young, of Monmouth, under whose care the girl had been for a few months. The mother informed him "that the child had always been peculiarly shaped, unusually short waisted, and the abdomen full, with a sensation of hardness. The catamenia first appeared at thirteen, and continued regular until some time during the past winter, when she missed two or three successive periods. At that time, the size increasing perceptibly and rapidly, she was first seen by Dr. Young. A thorough examination was refused, and only such a one permitted as could be obtained by palpation of the abdominal parietes. At a point corresponding with the position of the right ovary a tumor the size of a large orange was perceived, with evident fluctuation. From neither the mother nor child could any further facts be elicited. Under these circumstances hyd. potas., in combination with buchu and spts. ether. nit., were exhibited, and during that treatment the catamenia returned, though the tumor was apparently unaffected."

The tumor must have grown with unexampled rapidity during the time elapsing between the winter months and the following May, for at that time it was revealed most distinctly that the tumor was multilocular, containing at least seven cysts, which fact alone would make the case peculiarly unfavorable for the operation of puncture and iodized injections, for, besides the risk of severe constitutional irritation, there was the certain impossibility of reaching all the cysts, coupled with the uncertainty of obliteration of those sacs which could be reached by the trocar; while, on the other hand, the excellent general health of the patient, and the firm conviction that the case was not complicated with peritoneal adhesions, rendered the operation by incision and removal less hazardous in the performance and more certain in its results.

After due consideration, the operation of incision was resolved upon, and performed upon the 30th of May, by Dr. Byford, assisted by Drs. Young, Hamilton, McDill, and Taliaferro.

After having the room brought up to 98° Fahr., the patient was fully etherized and placed upon her back on the operating table; an incision through the abdominal walls, about midway between the umbilicus and symphysis pubis in the linea alba, three and a half or four inches long, exposed the tumor. This being done, I plunged a large trocar into it, and emptied one of the cysts of probably two pounds of thick albuminous transparent fluid. So soon as it ceased to run, I withdrew the trocar and introduced it into another sac, and so on, one after another, until eight cysts were evacuated, one of which contained eighteen pint cupfuls of liquid. The abdomen was now very much collapsed, and the tumor so much reduced in size that it was easily withdrawn through the incision. As it lay upon the table, the peduncle, which was about two inches broad, passed through the wound. Two strong silk ligatures were passed, one through each edge of the peduncle, near the tumor, in order to give us command of the stump after amputation. The écraseur was passed around the peduncle, between the ligatures and the ovary, and with it the mass was separated from its attachment. The stump was drawn between the lips of the incision, near its lower angle, and held in position by the two ligatures. Then silver pins, with steel points, were passed through the lips of the wound, passing near to, but not quite touching the peritoneum, and secured with thread as in hare-lip. A few fine silver-wire sutures closed up the wound superficially. Two of the silver pins used in transfixing the lips of the wound also passed through the stump of the detached tumor, and held its amputated margin a little above the level of the skin of the abdomen. A compress wet with cold water was placed upon the wound, all surrounded with a flannel binder, and the patient placed carefully back in bed. In about an hour she awoke from her sleep perfectly conscious, but entirely ignorant of what had occurred with respect to the operation, and expressed herself as perfectly comfortable.

The cysts and contents weighed thirty-nine pounds, and as it was an ordinary many-cysted mass, it would be unnecessary to give a particular description of it.

After the operation, the case, being some two hundred miles distant from Chicago, was left in charge of Drs. Taliaferro and Young. The case progressed favorably to the termination, with the exception of some considerable hemorrhage from the stump of the peduncle, which, however, was readily controlled by styptics. During the whole treatment, which continued until July fourth, at which time the case was discharged, the bladder acted naturally, the catheter not being needed once during the whole period. Dr. Byford concludes by summing up upon the mode of performing the operation.

I wish to make a very few remarks as to the mode of performing the operation.

It will be seen that there was no necessity of introducing the hand, or even the fingers, inside the peritoneal cavity. The whole mass was drawn through the wound by pulling upon the collapsed cysts, and there being no effusion of blood, or in fact anything else within the abdomen, the membrane was subjected to no rude handling, or other irritation, except what may have been produced by the entrance of air; and this was quite free, unavoidably. By drawing the stump through the wound, and placing the margin outside the abdominal cavity, the hemorrhage which often occurs, and did in this case from it, was external. The silver pins and wire produced as little irritation as anything else could. Although Dr. Young, in one of his notes, expressed regret that we had not ligated the stump, I would certainly not do so were I to operate again. The securing the stump in the wound made it impossible for the small sloughs, which are always thrown off from it, to irritate the peritoneum. It also, had we tied or clamped it, enabled us to avoid the inclusion of the ligature within the cavity. The écraseur is better for separating the tumor than a ligature, because, if the latter is used, the peritoneum must be strangulated and necessarily inflamed, and as inflammation spreads along this membrane with great facility, it may invade the abdominal cavity, and thus light up a fatal peritonitis. The hemorrhage that did occur, had it been suspected, could doubtless have been easily checked, and a little vigilance, I think, would render this operative procedure as safe as such an operation in the nature of things can be.—(*Chicago Medical Examiner*, July, 1860.)

